# Aluminum Phosphate Vaccine Adjuvant: Analysis of Composition and Size Using Off-Line and In-Line Tools

**DOI:** 10.1016/j.csbj.2019.08.003

**Published:** 2019-08-21

**Authors:** Carmen Mei, Sasmit Deshmukh, James Cronin, Shuxin Cong, Daniel Chapman, Nicole Lazaris, Liliana Sampaleanu, Ulrich Schacht, Katherine Drolet-Vives, Moriam Ore, Sylvie Morin, Bruce Carpick, Matthew Balmer, Marina Kirkitadze

**Affiliations:** a Sanofi Pasteur, Toronto, Ontario, Canada; b SGS Canada, Biopharmaceutical Services, Mississauga, Ontario, Canada; c Mettler Toledo AutoChem Inc, Columbia, MD, USA; d York University, Toronto, Ontario, Canada

**Keywords:** Aluminum phosphate adjuvant (AlPO_4_), Raman spectroscopy, Particle size distribution, Laser diffraction (LD), Fourier transform infrared spectroscopy (FTIR), Focused beam reflectance measurement (FBRM®), X-ray photoelectron spectroscopy (XPS), Process analytical technology (PAT)

## Abstract

**Purpose:**

Aluminum-based adjuvants including aluminum phosphate (AlPO_4_) are commonly used in many human vaccines to enhance immune response. The interaction between the antigen and adjuvant, including the physical adsorption of antigen, may play a role in vaccine immunogenicity and is a useful marker of vaccine product quality and consistency. Thus, it is important to study the physicochemical properties of AlPO_4_, such as particle size and chemical composition. Control of the vaccine adjuvant throughout the manufacturing process, including raw materials and the intermediate and final product stages, can be effectively achieved through monitoring of such key product attributes to help ensure product quality.

**Methods:**

This study focuses on the compositional analysis of AlPO_4_ adjuvant at the intermediate and final manufacturing stages using the off-line methods Fourier-Transform Infrared (FTIR) and Raman spectroscopy, X-ray Photoelectron Spectroscopy (XPS), and the in-line method Attenuated Total Reflectance (ATR). Particle size distribution of AlPO_4_ was measured off-line using Laser diffraction (LD) and in-line using Focused Beam Reflectance Measurement (FBRM®).

**Results:**

There was no observable difference in size distribution between the intermediate and final stage AlPO_4_ by off-line and in-line analysis, in both small- or large-scale production samples. Consistent peak shifts were observed in off-line and in-line infrared (IR) spectroscopy as well as off-line XPS for both small- and large-scale AlPO_4_ manufacturing runs. Additionally, IR spectroscopy and FBRM® for size distribution were used as in-line process analytical technology (PAT) to monitor reaction progress in real-time during small-scale AlPO_4_ manufacturing from raw materials. The small-scale adsorption process of a model protein antigen (Tetanus toxoid) to AlPO_4_ adjuvant was also monitored by in-line ReactIR probe.

**Conclusion:**

This study demonstrated that in-line PAT can be used to monitor particle size and chemical composition for the various stages of adjuvant manufacturing from raw materials through intermediate to final adjuvant product stage. Similar approaches can be utilized to help assess lot-to-lot consistency during adjuvant manufacturing and vaccine product development. Moreover, the use of in-line PAT is highly conductive to advanced manufacturing strategies such as real-time product release testing and automated processes of the future.

## Graphical Abstract

**Figure:**
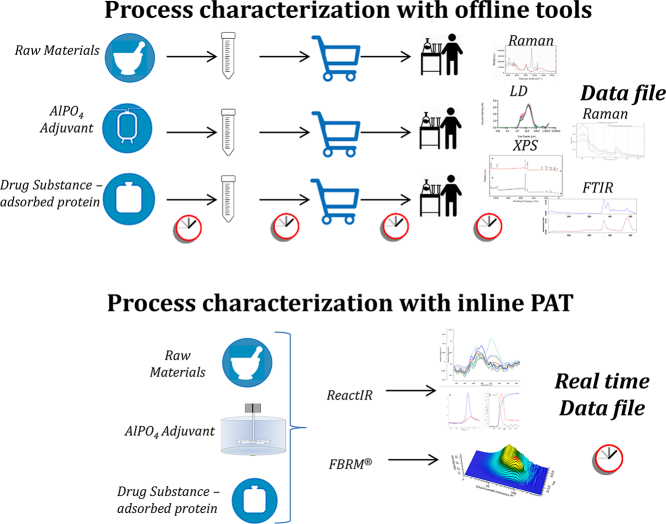

## 1 Introduction

Many vaccines require formulation of antigens with adjuvants for optimal immunogenicity and efficacy. Aluminum-based adjuvants such as AlPO_4_ have been used for many years in various human vaccines to enhance immune response. The immunostimulatory effect of aluminum-containing adjuvants is influenced by the adsorption process of antigen to adjuvant [1]. Antigens can adsorb to AlPO_4_ adjuvant by ligand exchange, electrostatic, hydrophobic, or van der Waals interactions [2]. The importance of adsorption of antigens on the surface of aluminum adjuvants has been reviewed in the published literature [3]. The precise mechanisms of vaccine immunogenicity enhancement by aluminum adjuvants are still not well understood; thus the utility of any candidate adjuvant to a given vaccine antigen formulation cannot be predicted a priori and must be established on a case-by-case basis by means of clinical efficacy trials. It is therefore important to establish, as early as possible in the clinical development phase, quality attributes for vaccine active components including adjuvants.

Through technological advancement, biochemical and biophysical analyses are now feasible for real-time process monitoring using in-line PAT. This methodology typically incorporates the use of probes that are directly inserted into manufacturing reactors, containers, and connectors. In-line PAT offers advantages such as facilitating real-time release testing (RTRT), reducing time delays from testing in off-line quality control laboratories, digitizing batch release records, and helping to ensure lot-to-lot consistency through automation and streamlining of the process [4]. The monitoring of quality attributes by in-line PAT throughout vaccine developmental phases and through product licensure will also help ensure that the eventual marketed product is consistent with the candidate for which safety and efficacy was demonstrated in the clinic. Moreover, using efficient, robust, and informative in-line analytical tests to monitor key product attributes will help accelerate new product development as well as life cycle management (LCM) for marketed vaccines. Information on adjuvant structure and size distribution at various process stages, up to and including the adsorbed antigen stage may be useful to determine the effect of adjuvant on antigen structure and vaccine immunogenicity [5].

The size distribution and crystallinity of AlPO_4_ varies depending on the preparation [6,7]. This study focuses on the characterization of in-house AlPO_4_ adjuvant using particle sizing technology, IR and Raman spectroscopy to examine P—O bond shifts, and XPS for elemental analyses. In addition to the more traditional off-line methods, the particle size and P—O bond shifts of AlPO_4_ were assessed by in-line PAT methods using probes. The purpose of this study is to demonstrate that in-line PAT is also comparable with advanced manufacturing strategies including real-time release testing and increased automation. Hence, having the potential potential to streamline the manufacturing process by generating results in real-time, allowing process decisions to be made faster.

Intermediate (in-process) and final (product) manufacturing stages of AlPO_4_ from large-scale production runs were analyzed using off-line and in-line tools to monitor differences in quality attributes, specifically particle size and P—O bond shifts. In addition, off-line XPS compositional analysis was deployed to examine differences in the elemental levels of Al, P, and O in intermediate and final AlPO_4_ stages. The FBRM® and ATR in-line probes were used to monitor changes in size distribution and composition of AlPO_4_, respectively, during a small-scale precipitation reaction from raw materials. Small-scale adsorption of Tetanus toxoid to final stage AlPO_4_ adjuvant was monitored by IR spectroscopy using the in-line ATR probe. Composition of the solid raw materials were characterized off-line using Raman spectroscopy (Fig. S1) whereas the in-line ReactIR probe was used to observe the mixing of the solubilized raw materials. See Table 1 for a summary of the off-line and in-line tools that were used to characterize the material attributes of all the examined AlPO_4_ runs.

**Table 1:** Summary of off-line and in-line methods.

Analyte, scale	Raw materials	Large-scale intermediate and final stage AlPO_4_	Small-scale intermediate and final stage AlPO_4_	Small-scale Tetanus toxoid adsorption to final stage AlPO_4_
Number of samples	6	6	3	1
Laser Diffraction, LD	–	√	–	–
Fourier transform Infrared Spectroscopy, FTIR	–	√	√	√
Raman Spectroscopy	√	√	–	–
X-ray Photoelectron Spectroscopy, XPS	–	√	–	–
Focused Beam Reflectance Measurement, FBRM®	–	√	–	–
Infrared in-line probe, ReactIR	–	√	√	√
Raman in-line probe, ReactRaman	√	–	–	–

Note: “√” indicates that method was performed, whereas “-” indicates that method was not used.

## 2 Materials and Methods

Table 1 summarizes the off-line and in-line tools that were used to characterize the material attributes of the relevant steps of the small and large scale AlPO_4_ manufacturing runs. Three lots of large-scale intermediate (in-process) and final (product) stage AlPO_4_ were examined, while all small-scale AlPO_4_ was produced in one precipitation reaction. In this study, the small scale was of 100 mL, while large scale was of approximately 2000 times greater than small scale. The samples from large-scale batches were obtained by dispensing into 50 mL tubes from a larger container. For the in-line measurements, the samples were loaded in the Easy Max, stirred, and examined by submerging FBRM® and ReactIR probes into the Easy Max reactor vessel. Table 2 summarizes the material attributes of the AlPO_4_ adjuvant measured by a panel of techniques.

**Table 2:** Material attributes of AlPO_4_ adjuvant.

Method	Material attributes
LD	Particle size
FTIR spectroscopy	P-O Bond Shift
Raman spectroscopy	P-O Bond Shift
XPS	Surface elemental composition
FBRM®	Particle size

### 2.1 Off-Line Analyses

Aluminum phosphate adjuvant samples were manufactured in-house by Sanofi Pasteur Canada, Toronto site from the raw materials aluminum chloride hexahydrate (AlCl_3_·6H_2_O) and sodium phosphate tribasic dodecahydrate (Na_3_PO_4_·12H_2_O) in large-scale reactors. The solid raw material salts were individually dissolved in Milli-Q water to form clear colourless solutions. Reaction of these two solutions upon mixing formed the white, opaque AlPO_4_ adjuvant suspension. Intermediate (in-process) and final (product) stage AlPO_4_ samples were obtained from the Sanofi Pasteur Canada, Toronto site and stored at 2–8 °C until measurement.

### 2.2 Laser Diffraction (LD)

The particle size distribution of AlPO_4_ adjuvant was determined using the Mastersizer 3000 instrument (Malvern Instruments Ltd. UK), operating in a dynamic range of 0.01 to 3500.00 μm. Particle size distributions of suspensions were quantitatively determined by measuring the angular variation in intensity of the light scattered from a laser beam passing through a dispersed particulate sample in Milli-Q water. Measurements were recorded at 1.5% laser obscuration, and stirring speed of 1000 rpm. Large particles scatter light at small angles, and small particles scatter light at large angles. The Mastersizer 3000 software (Malvern Instruments Ltd. UK) uses the angular scattering intensity data to calculate the size distributions of the particles responsible for creating that scattering pattern using the Mie theory of light scattering. The reportable value, Derived Diameter (Dv), is the particle size (in μm) for a specific percentile of the cumulative size distribution. No sample preparation was required. Five consequtive measurements with % obscuration of greater than 1.5 were used for calculation of the Dv. Particles in liquid suspensions were measured using the built-in “non-spherical” option within the software, and an average Dv10, Dv50, and Dv90 value of 5 measurements were reported. These values are the mean diameters at which the given percent (10, 50, or 90) of particles in the sample is smaller than the reported value.

### 2.3 Raman Spectroscopy

Raman spectroscopy was performed using the i-Raman Plus® portable Raman system (B&W Tek Inc., Newark, DE) equipped with a 785 nm laser. The spectra were collected from 0 cm^−1^ to 3200 cm^−1^. Data acquisition and analysis were performed using the BWSpec4 software system (B&W Tek Inc., Newark, DE), whereby baseline correction was applied for all measured spectra. The instrument reports the intensity as a function of Raman shift which is displayed in a graph. The background vibrations were corrected by acquiring a dark scan in the absence of the laser before each measurement. This was then automatically subtracted from each measurement. These spectra were then re-plotted using SigmaPlot.

To begin Raman analysis, the samples of AlPO_4_ at intermediate (in-process) and final (product) manufacturing stages were first centrifuged at 2000 ×*g* for 4 min to obtain a precipitate. The supernatant was discarded and the pellets were transferred evenly on weighing plates. These weighing plates were then placed in a clean transparent box to prevent contamination from foreign materials. The samples on the plates were left to air dry at room temperature (22–23 °C) overnight. The dried AlPO_4_ adjuvant was then pulverized using a spatula and transferred to a quartz cuvette for analysis by Raman spectroscopy. In addition to air drying the AlPO_4_ at room temperature, drying was also perfomed using the 37 °C incubator analyzed by Raman spectroscopy. However, no differences were noted in the resultant spectrum. Solid AlCl_3_ and Na_3_PO_4_ raw materials were also analyzed by Raman spectroscopy in a similar way by transferring sample into a quartz cuvette and placing it in the cuvette holding assembly of the Raman spectrometer.

### 2.4 Fourier Transform Infrared Spectroscopy (FTIR)

FTIR spectroscopy was performed using a Vertex 70 FTIR Spectrometer (Bruker Optics, Bremen, Germany), equipped with a cryogenically-cooled mercury-cadmium-telluride (MCT) detector and BioATRII sampling accessory. No sample preparation was required. A sample volume of 20 μL AlPO_4_ was loaded onto the sample cell of the BioATRII. The spectra were collected at a resolution of 0.4 cm^−1^ at 25 °C with a wavenumber accuracy of 0.01 cm^−1^ at 2000 cm^−1^. The samples were allowed 1 min to stabilize on the ATR crystal. Buffer (Milli-Q water) and samples were then analyzed, with each sample measurement averaging 200 scans. Data acquisition and analysis were performed using the OPUS 6.5 software (Bruker Optics, Bremen, Germany). OPUS automatically subtracted the background (buffer) signal from the sample to produce the spectrum of the analyte. All measurements were carried out at 25 °C using the Haake DC30/K20 temperature controller (Karlsruhe, Germany). After acquiring the FTIR spectra, the baseline was corrected by removing the scattering signal using the OPUS software. *Re*-plotting of the spectra was performed using SigmaPlot. FTIR was not used to analyze solid materials due to the use of a scratch-prone ATR crystal.

### 2.5 X-ray Photoelectron Spectroscopy (XPS)

X-ray photoelectron spectroscopy (XPS) spectra were collected using a PHI Quantera II photoelectron spectrometer (Physical Electronics) with an Al Kα monochromatic X-ray source (250 W, hv = 1486.6 eV of photons), a hemispherical analyzer and a multichannel detector. The vacuum in the analytical chamber was approximately 6.7 × 10^−9^ Torr during measurements. The elemental composition was determined from survey spectra were recorded at a pass energy of 280.0 eV. The high-resolution spectra of Al atomic orbitals 2p, O atomic orbitals 1 s, P atomic orbitals 2p, were recorded at a pass energy of 26.0 eV. The binding energy scales were charge referenced to the Na 1 s peak at 1072 eV. The measurements were performed using standard XPS procedure with electron takeoff angles of 15° and 45° with respect to the sample surface. The curve-fitting analysis were performed using a generalized Lorentzian line shape LA (1.53,243) curve fitting function in the CasaXPS software. U 2 Tougaard background subtraction method was used for the fitting.

### 2.6 In-Line Analyses

For the evaluation of in-line process analysis, a small-scale reaction of AlCl_3_ and Na_3_PO_4_ raw materials was used to mimic the manufacturing process of AlPO_4_ adjuvant. Solid AlCl_3_ and Na_3_PO_4_ was first each dissolved in Milli-Q water. The AlCl_3_ and Na_3_PO_4_ salt solutions were then sequentially added to the EasyMax 102 table-top reactor (Mettler Toledo Inc., USA) for mixing. For this experiment, the AlCl_3_ solution was first added to the reactor first, followed by addition of the Na_3_PO_4_ solution using an automated syringe pump. The reaction progress from the addition of raw materials to the completion of AlPO_4_ precipitation was monitored in real-time by in-line particle sizing, and IR and Raman spectroscopy probes. Size distribution profiles and IR and Raman spectra were recorded at different time intervals throughout the reaction. In-line ReactIR probe was also used to characterize Tetanus toxoid in a small-scale overnight adsorption reaction to final AlPO_4_. Additionally, samples of large-scale intermediate and final stages of AlPO_4_ were analyzed using the in-line probes. For the purpose of assessing the feasibility of in-line process analysis in biophysical characterization, in-line particle size data, and IR and Raman spectra of intermediate and final AlPO_4_ were compared to those obtained from off-line analysis.

### 2.7 Focused Beam Reflectance Measurement (FBRM®)

Real-time particle size data was determined using the ParticleTrack probe (Mettler Toledo Inc., USA). This probe was directly inserted into the EasyMax 102 reactor where particles in suspension could flow easily across the sapphire window. Equipped with FBRM® technology, a laser beam is directed down a set of optics along the probe and is focused to a tight beam spot at the window. The rotating optics focuses the beam, which then rapidly scans across particles as they flow past the window. The resulting light scattering pattern from the particles is detected by the probe and used to calculate the chord length, or distance across each particle. The reportable value in FBRM® is the chord length at percentile C, which is C50 (in μm) in this case. This value corresponds to the Dv50 value as reported for LD. The real-time chord length distribution was monitored using iC FBRM™ ParticleTrack software (Mettler Toledo Inc., USA).

### 2.8 Infrared (IR) Spectroscopy (In-line)

IR spectra were recorded using the ReactIR 702 L (Mettler Toledo Inc., USA). This probe is equipped with an Attenuated Total Reflectance (ATR) sensor that measures the changes of the IR beam as it is internally reflected upon contact with the sample. The resulting beam will be attenuated in the regions of the IR spectrum where the sample absorbs energy. This attenuated beam returns to the ATR crystal and exits the opposite end, and is directed to the detector. The software iCIR (Mettler Toledo Inc., USA) was programmed to collect IR spectra at set time intervals throughout the AlPO_4_ precipitation reaction. All spectra were plot in absorbance units.

Prior to the AlPO_4_ precipitation reaction, an IR spectrum was recorded for each starting material (AlCl_3_ and Na_3_PO_4_ salt solutions) and product (AlPO_4_ that was previously manufactured large-scale). One distinct peak from each spectrum was assigned to the material. Additional attention was given to these select peaks from the AlCl_3_ solution and AlPO_4_ spectra. The iCIR software was then used to monitor the real-time spectral changes occurring in the reaction mixture as AlPO_4_ was precipitated from raw materials. More specifically, the changes in the P—O bond shift was observed. A peak height (normalized AlCl_3_ and AlPO_4_ peaks) versus time plot was used to give qualitative information on the reaction progress and relative quantities of reactant versus product. In this study, normalization refers to assigning the reactant peak as 0% and the final product peak as 100% of the height.

### 2.9 Raman Spectroscopy (In-Line)

Raman spectra were recorded using the ReactRaman in-line probe (Mettler Toledo Inc., USA.). Similar to the off-line method, this in-line probe is also equipped with a laser that serves as the excitation source to induce Raman scattering. The energy from the laser is transmitted to the sample surface via fibre optic cables. The resulting Raman signal is then filtered in the fibre optic cables to eliminate Rayleigh and Anti-Stokes scattering. The remaining Stokes scattered light is passed on to a dispersion element and a Charge-Coupled Device (CCD) captures this light and records the results in the Raman spectrum in the iC Raman 7 software (Mettler Toledo Inc., USA). A Raman spectrum for each salt solution (AlCl_3_ and Na_3_PO_4_) was collected prior to the AlPO_4_ precipitation reaction. This demonstrated the differences in characteristic peaks positions in each raw material in the solid versus solubilized forms.

## 3 Results

### 3.1 Off-Line Analysis

Two attributes of AlPO_4_ that are discussed in this paper are the particle size distribution and P—O bond shifts of AlPO_4_ adjuvant. P—O bond shifts are dependent upon the overall structure of the P—O containing raw material, intermediate, and final adjuvant, and thus can be used to monitor the progress of the adjuvant manufacturing process and its completeness. The difference in Al, P, and O content between intermediate and final adjuvant production stages was also assessed as an additional diagnostic tool.

**Fig. 1:**
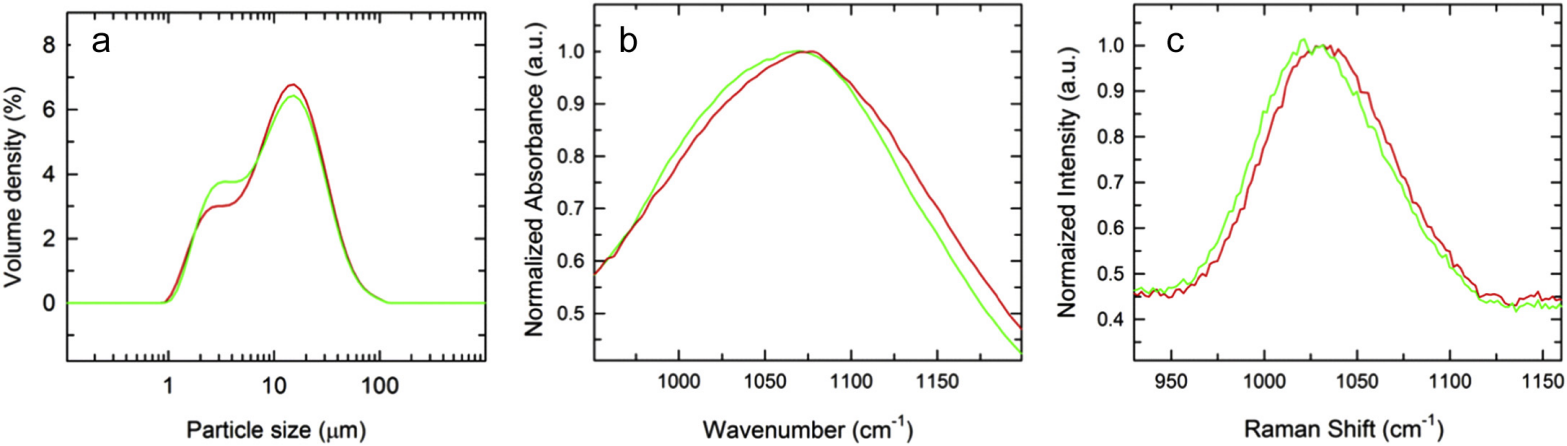
(a) Particle size distribution profiles of intermediate (green trace) and final (red trace) AlPO_4_ measured by LD; (b) FTIR and (c) Raman spectra of intermediate (green trace) and final (red trace) AlPO_4_ manufactured at large scale.

LD demonstrated a prominent peak at 11 μm and a shoulder at around 2 μm. See Fig. 1a for comparison of the size distribution profiles between intermediate and final AlPO_4_ adjuvant manufactured at large scale. There was no observable change between the intermediate and final stage adjuvant in terms of overall particle size distribution; both size distribution profiles showed the majority of particles in the population with an average size of 11 μm. However, a minor change in the ratio of the shoulder height (volume density %) was observed between intermediate and final AlPO_4_. FTIR showed a 9 cm^−1^ shift in the phosphate group P—O stretch in the final AlPO_4_ spectrum. The peak of the P—O stretch in the intermediate AlPO_4_ was around 1067 cm^−1^, while the same peak in the final AlPO_4_ shifted to 1076 cm^−1^ (Fig. 1b). Similarly, Raman spectroscopy also revealed a shift in the P—O stretch peak of 10 cm^−1^ (1024 cm^−1^ to 1034 cm^−1^) from the intermediate to final stage of AlPO_4_ adjuvant (Fig. 1c). Solid samples of AlCl_3_ and Na_3_PO_4_ raw materials were analyzed by Raman spectroscopy to identify key spectral features (Fig. S1). Na_3_PO_4_ showed prominent Raman peaks for the phosphate group at 412 cm^−1^, 548 cm^−1^, and 942 cm^−1^. AlCl_3_ showed AlCl_3_ stretch at 560 cm^−1^, Al—O stretch at 425 cm^−1^, and hydrated AlCl_3_ at 520 cm^−1^. As observed with the in-line methods, the solubilized raw materials salts have different distinct peaks. Unlike AlPO_4_ which showed only the P—O stretch as a prominent feature in the Raman spectrum, raw materials such as AlCl_3_ and Na_3_PO_4_ showed rich spectral features. Solid Na_3_PO_4_ showed prominent Raman peaks for the phosphate group at 412, 548 and 942 cm^−1^ while solid AlCl_3_ showed Al—Cl stretch at 560 cm^−1^, Al—O stretch at 425 cm^−1^ and hydrated AlCl_3_ around 520 cm^−1^ (Fig. S1). These attributes could be monitored for raw materials testing, in light of the potential impact on AlPO_4_ adjuvant composition or other product characteristics.

**Fig. 2:**
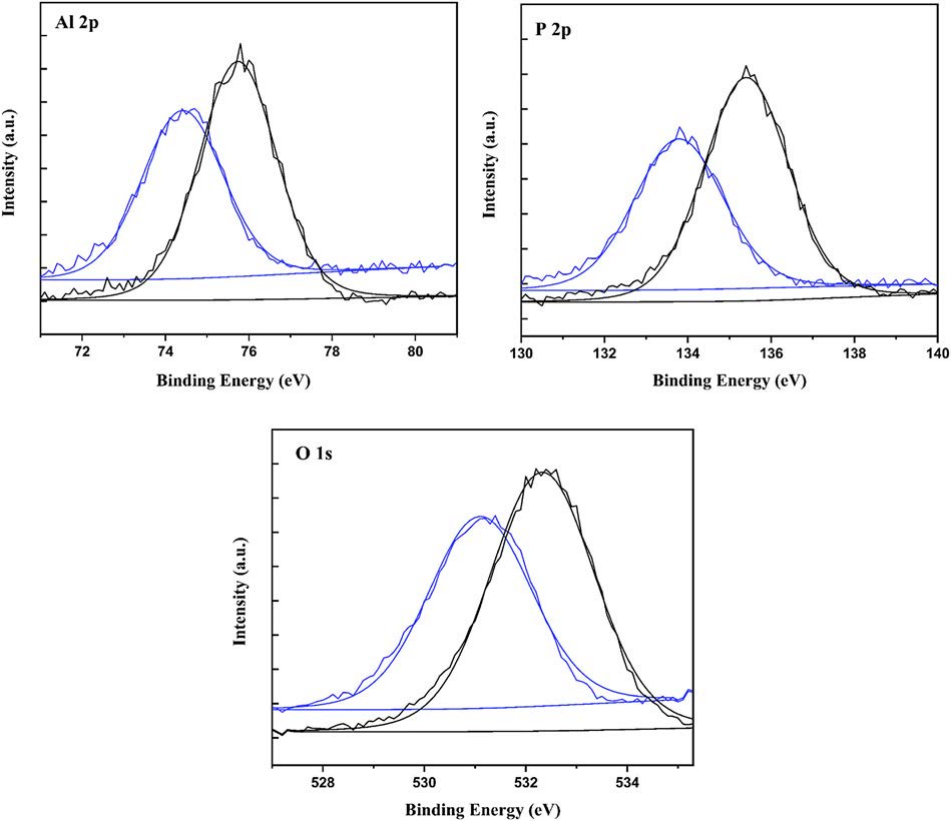
High resolution XPS spectra collected with the 15° take-off angle for Al 2p, P 2p, and O 1 s of intermediate (blue trace) and final (black trace) AlPO_4_ sample. Smooth curves represent the fitting. The horizontal lines on the graph represent the baseline used for fitting.

XPS analysis was performed using two different electron take-off angles (15° and 45°) to determine the elemental composition of the intermediate and final AlPO_4_ suspension material stages (Fig. S2). The same six elements were detected for both the intermediate and final AlPO_4_ stages (Table S1). The P/Al ratio is close to 1, which was the same as observed previously by NMR [6]. The high-resolution spectra for O1s, Al 2p, and P 2p were examined. For all three photoelectrons, there was a shift to higher binding energy for the final AlPO_4_ versus intermediate (Fig. 2). As summarized in Table S2, the binding energy increase ranged between 1.22 eV (for O 1 s) and 1.61 eV (for P 2p) in the 15° take-off angle experiment. For the 45° angle experiment, the energy increases were smaller in magnitude (less than 1 eV in all cases), however the increase was consistently observed for all three photoelectron spectra (Table S2). The observed shift can be attributed to differences in coordination of Al and P in the materials. During the transition from intermediate to final stages the Al and P in amorphous materials such as AlPO_4_, can experience changes in their coordination giving rise to changes in binding energies [8,9]. Therefore the observed shifts could be due to an increase in coordination and ordering of the structure of the final AlPO_4_ material versus that of the intermediate stage. The difference between the intermediate and the final AlPO_4_ stages is consistent with IR and Raman spectroscopy results. Apart from its application in vaccine formulation as an adjuvant, AlPO_4_ is also used in the synthesis of chemical catalysts [10]. For these products, assays such as XPS are widely used to also characterize the amorphous and crystalline phases of AlPO_4_ [9].

### 3.2 In-Line Analysis

**Fig. 3:**
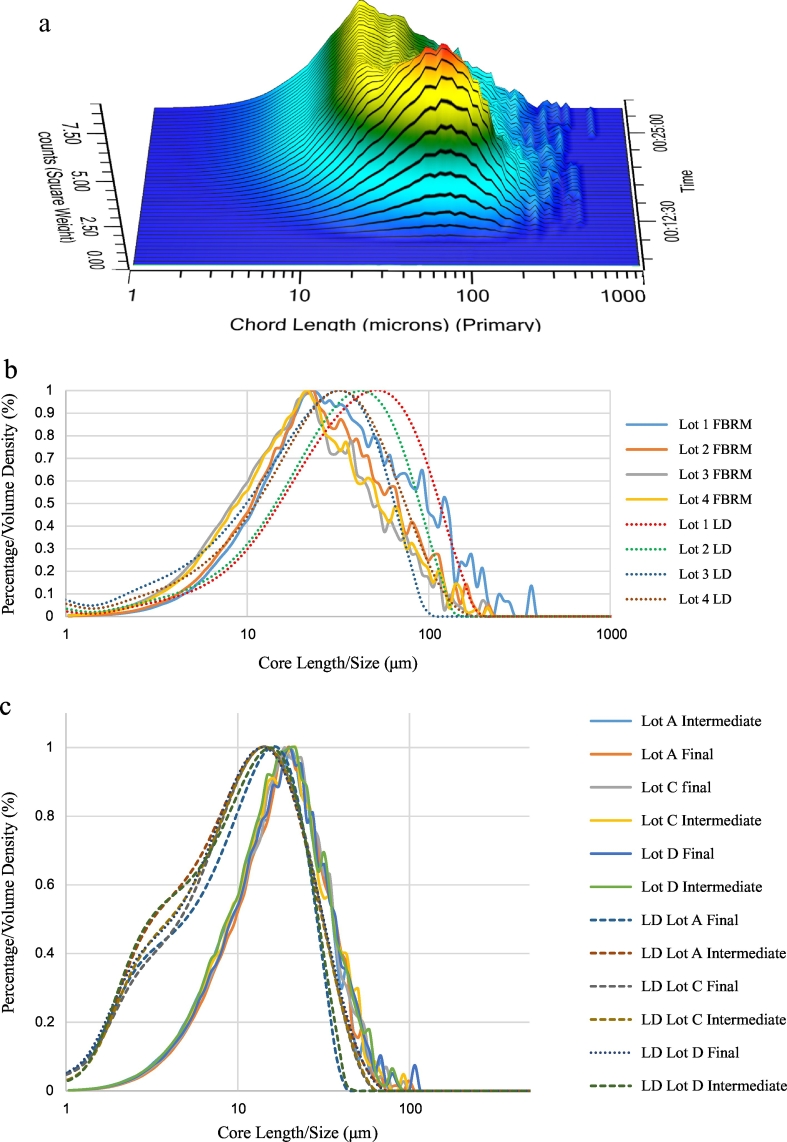
(a) AlPO_4_ precipitation reaction monitoring in real-time (b) Overlay of four AlPO_4_ intermediate lots size distribution profiles by LD (orange, dotted) and FBRM® (blue, solid); each lot was prepared by mixing AlCl_3_ and Na_3_PO_4_ raw materials in the EasyMax reactor (c) The size distribution of manufacturing scale AlPO_4_ by LD, intermediate and final (dotted), and by FBRM®, intermediate and final (solid).

FBRM® method was used to monitor particle size distribution of the reaction mixture in real-time during AlPO_4_ precipitation from AlCl_3_ and Na_3_PO_4_ (Fig. 3a). Several trials of the reaction were run using different mixing speeds and dosing rates of Na_3_PO_4_. Particle size analyses of these small-scale AlPO_4_ runs did not show observable differences between runs, regardless of mixing speed or dosing rate (data not shown). Fig. 3b for shows an overlay of FBRM® and LD particle size profiles for the small-scale runs. Based on four runs, the average particle size of AlPO_4_ that was produced in samples from the small-scale reactions was approximately 25 μm and 21 μm as determined by LD and FBRM® respectively. The average particle size for the same small-scale samples analyzed by off-line LD was slightly lower, but the overall profiles were similar for both methods (Fig. 3b). AlPO_4_ samples from large-scale manufacturing runs (same samples as analyzed using the off-line methods) were also tested using the in-line FBRM® probe. These three intermediate and three final AlPO_4_ product samples had an average particle size of approximately 11 μm by FBRM®. The average particle size for the same lots as determined by LD was slightly lower, while the overall shapes of the distributions were similar, although the LD profiles showed a shoulder peak at approximately 2–3 μm (Fig. 3c). As observed with off-line LD, in-line FBRM® showed no differences in size distribution between intermediate and final AlPO_4_ product (Fig. 3c, solid traces). For small scale lots once the AlPO_4_ was formed its particle size remained the same as shown for the small scale lots in Fig. 3b. Same is true for the large-scale lots that are shown in Fig. 3c. The aliquots of the large-scale lots were tested by both LD and FBRM®. Therefore, from the particle size perspective, FBRM® method is deemed suitable to measure the size the AlPO_4_.

The differences in the results obtained by LD and FBRM® are due to the experimental setting used for each method. For LD there is an approximately 100 fold dilution of AlPO_4_, which makes the small particle groups visible or creates small particle groups due to dilution. Whereas for FBRM® is performed for the AlPO_4_ material “as is” without any dilutions, and thus appears to be more valuable for the characterization AlPO_4_ manufacturing process.

**Fig. 4:**
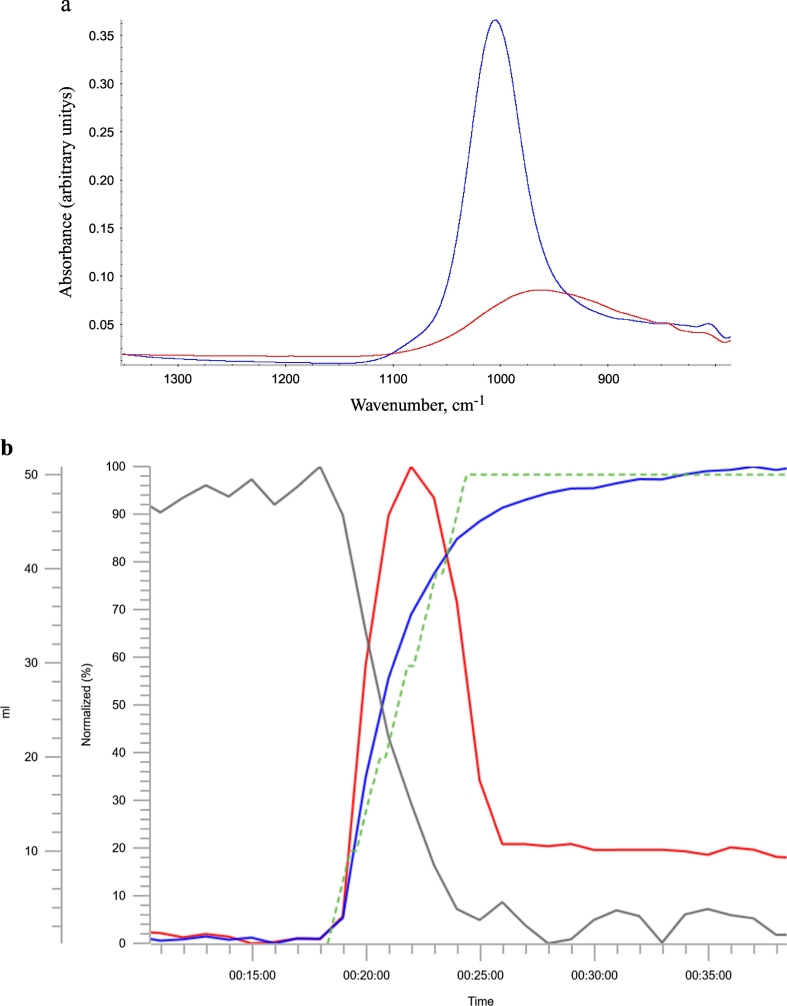
(a) The inline IR spectral overlay of AlCl_3_ (red trace) and Na_3_PO_4_ (blue trace); (b) Inline IR monitoring of AlPO_4_ adjuvant formation during the small-scale precipitation reaction. The normalized IR peak height corresponding to AlCl_3_, AlPO_4_ transient intermediate are represented by the solid gray, solid blue, and solid red traces respectively. The dotted green trace represents the volume of Na_3_PO_4_ (in mL) added.

In-line ReactIR probe and Raman analysis provided spectral information on the raw materials prior to AlPO_4_ precipitation. FTIR spectra of AlCl_3_ and Na_3_PO_4_ solutions showed major peaks at 960 and 1004 cm^−1^, respectively (Fig. 4a). These peaks were identified as key spectral features and were subsequently used to monitor the precipitation reaction. Raman peaks were similarly assigned to the same raw materials solutions. The peaks of interest were at 524 cm^−1^ for AlCl_3_ solution, and 937 cm^−1^ for Na_3_PO_4_ solution (Fig. 4S). It was found that IR spectroscopy can be used to analyze liquid samples, whereas Raman spectroscopy is more sensitive to solid samples (data not shown). Therefore it was decided to focus on the use of in-line ReactIR probe to monitor the AlPO_4_ precipitation reaction in real time.

The key spectral features identified from the in-line ReactIR analysis of the raw material solutions were tracked according to changes in their peak position and height throughout the small-scale reaction. This gave information on the progress of the reaction. In this study, the IR peaks of interest were at 933 cm^−1^ for AlCl_3_, 1086 cm^−1^ for final AlPO_4_, and 1155 cm^−1^ for intermediate AlPO_4_. It is important to note that solid and solubilized raw materials will have different wavenumbers (Fig. S1). Fig. 4b plots the changes in the normalized heights (in %) of these peaks over time, along with the volume (in mL) of Na_3_PO_4_ added to the solution was followed immediately by a decrease in AlCl_3_ and increase in AlPO_4_ peak heights. The transient intermediate AlPO_4_ peak was a key indicator of the reaction progress as it was observed only upon the addition of Na_3_PO_4_ but had dissipated prior to completion of the reaction. The plateau observed at the end of precipitation indicated there was no longer any change in the reactants and product. Thus, the reaction had reached completion.

**Fig. 5:**
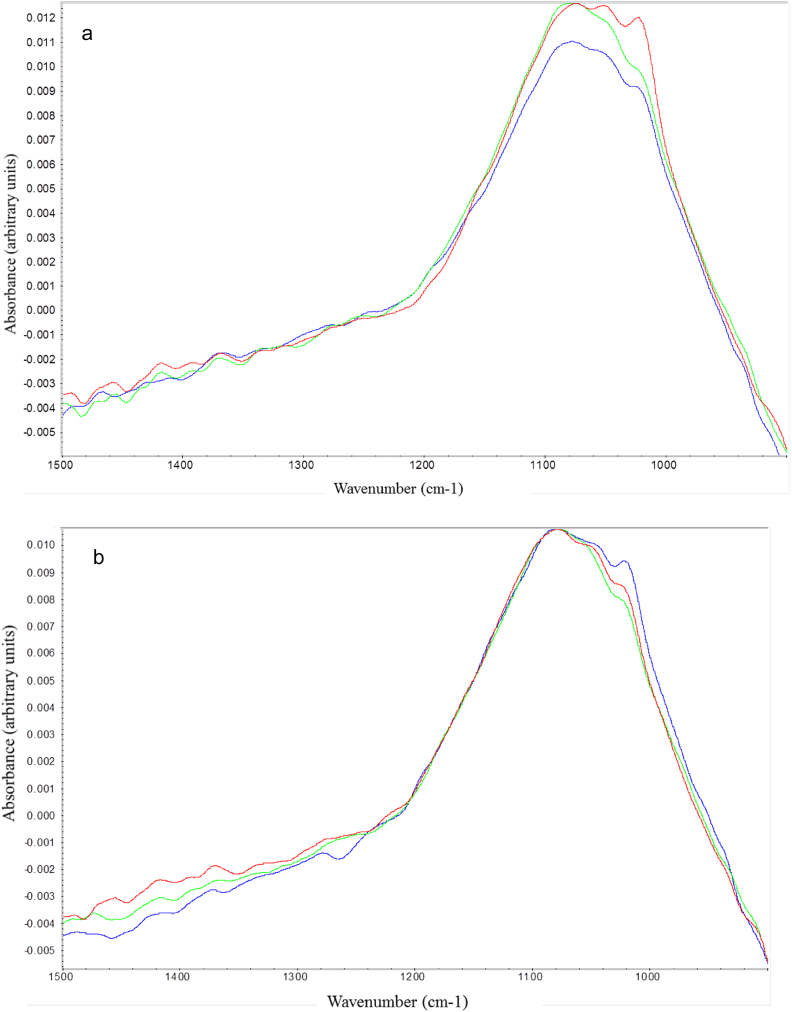

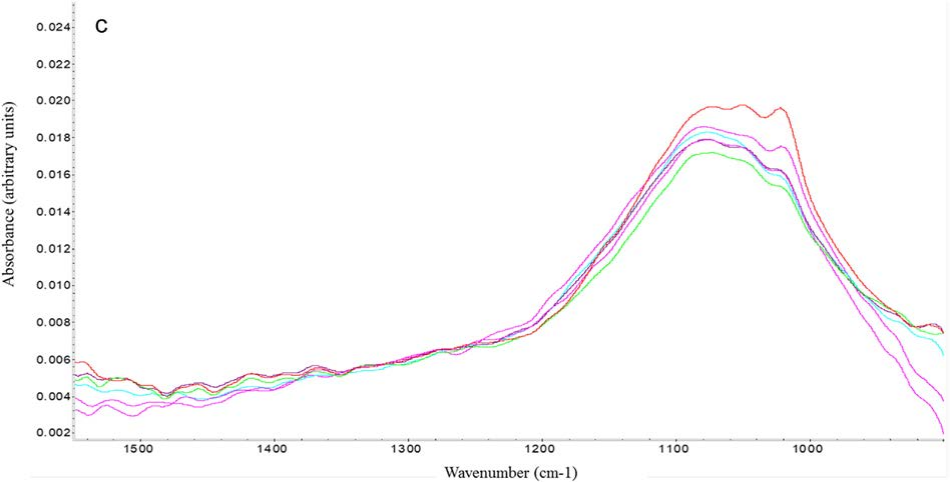
IR Spectra of (a) intermediate and (b) final AlPO_4_ (small-scale) using inline analysis; (c) IR Spectra of intermediate (red, magenta, and purple with a broad plateau) and final (green, blue, and pink with a narrow plateau) stage AlPO_4_ (large-scale) using inline analysis.

In the small-scale AlPO_4_ precipitation reaction, a shift in wavenumber was observed at peak positions 1021 cm^−1^ and 1081 cm^−1^ from intermediate to final AlPO_4_ during the precipitation reaction. A similar result was also observed when AlPO_4_ samples from large-scale manufacturing were analyzed in-line: an increase in wavenumber of 4 cm^−1^ was observed at position 1067 cm^−1^ from intermediate to final AlPO_4_ (Fig. 5b). As this spectral region was initially assigned to the phosphate group of AlPO_4_, this shift suggested the presence of a stronger P—O bond in the final AlPO_4_ product (Fig. 1b). Off-line FTIR analysis of AlPO_4_ from large-scale production also demonstrated an increase of 9 cm^−1^ from the intermediate to final stage at a similar peak position (Fig. 1a). Thus, a consistent data trend was established between in-line and off-line analysis for monitoring IR spectroscopy of intermediate and final AlPO_4_ for both small- and large-scale samples.

**Fig. 6:**
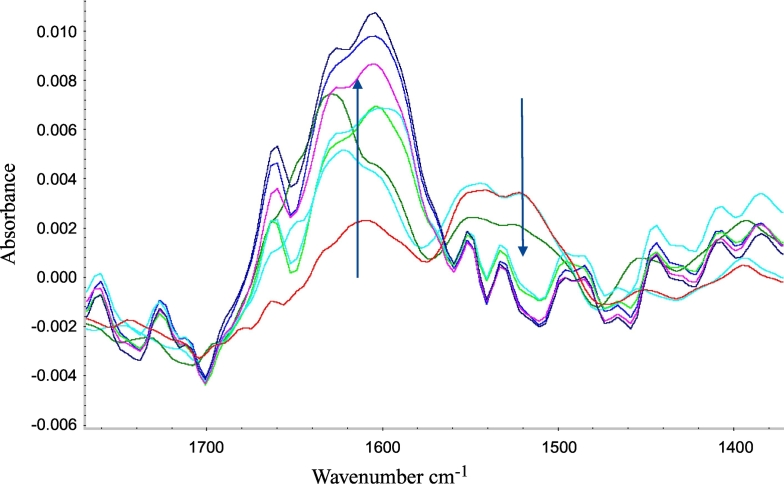
IR spectral overlay during inline monitoring of Tetanus Toxoid adsorption to final AlPO_4_ adjuvant. Red trace represents Tetanus Toxoid IR spectrum at the beginning of adsorption reaction. Amide II peak decreases during adsorption (downward arrow), while Amide I peak becomes more prominent (upward arrow), which is consistent with FTIR spectrum of Tetanus Toxoid reported previously [5].

Previously, the following characteristics such as particle size distribution, morphology, elemental analysis, and secondary structure were reported for the adsorbed Tetanus Toxoid, Diphtheria Toxoid, Pertussis Toxoid, Pertactin, Fimbriae 2,3, and Filamentous Haemagglutinin [5]. Both particle size and morphology is driven by AlPO_4_, whereas protein-antigen adsorption results in changes of secondary structure content of the protein. Therefore in this study, Tetanus Toxoid was used as model to examine the small-scale adsorption process of protein antigen to AlPO_4_ final adjuvant was also monitored in real-time by in-line ReactIR probe. The purpose of this analysis was to monitor conformational changes in the protein expected to be associated with interactions with the adjuvant, and thus follow the overall course of the adsorption process. The Amide *I* region of the antigen protein spectrum was of particular interest in this study as changes in its peak height were expected to be diagnostic of antigen adsorption to AlPO_4_ adjuvant [5]. The peaks of interest were at 1663 cm^−1^ corresponding to antigen adsorbed to AlPO_4_, and 1518 cm^−1^ representing antigen not adsorbed to AlPO_4_. The changes in height of these two peaks were monitored to give qualitative information on the relative concentrations of adsorbed versus non-adsorbed antigen. Fig. 6 shows an overlay of IR spectra recorded in real time at various timepoints throughout the course of the adsorption reaction. The results showed a general increase in peak height at 1663 cm^−1^ and general decrease in peak height at 1518 cm^−1^ over period of 16 h. This was an indication that the amount of adsorbed Tetanus Toxoid was increasing while the amount of non-adsorbed antigen was decreasing. The spectral shift as a result of adsorption is similar to that observed using off-line FTIR and is indicative of the increase in secondary structure content [8]. In-line ReactIR probe is thus able to give qualitative information during the AlPO_4_ precipitation and antigen adsorption processes in order to monitor progress and completeness.

## 4 Discussion

Aluminum adjuvants such as AlPO_4_ are commonly used in vaccine products to stimulate the immune response against antigens, and are required for optimal immunogenicity and efficacy of many vaccines. Different antigens adsorb to different extents onto the surface of adjuvants and can undergo structural changes that may stabilize or destabilize antigens. Jones et al. showed that lysozyme, ovalbumin and bovine serum albumin (BSA) experience a decrease in unfolding temperature upon adjuvantation [11]. In another study, tuberculosis vaccine candidate antigen protein became more stable upon adsorption onto a different type of adjuvant [12]. This study demonstrates the importance of compositional characterization of adjuvants, such as the P—O bond shift of AlPO_4_, which could be used as a material attribute of AlPO_4_ adjuvant. Decreases in amide II peak in the IR spectrum of Tetanus Toxoid following adsorption to AlPO_4_ have thus been observed by both in-line (this study) and off-line [5] measurements. The adsorption of antigens to adjuvants and the conformation of antigens in the presence of aluminum adjuvants can affect the vaccine immunostimulatory response [1]. Therefore, the profile of the manufactured AlPO_4_ adjuvant itself, along with the properties of the adsorbed antigens is important to monitor from the perspective of vaccine product consistency.

Particle size of an adjuvant can determine such properties as the available surface area for antigen adsorption. This can in turn impact antigen conformation and potentially presentation of functional epitopes to the immune system of immunized individuals. According to empirical studies, the optimal particle size for AlPO_4_ adjuvant is approximately 10 μm [13] and in close agreement with the average size distribution reported in this study by LD of approximately 11 μm (Fig. 1a). The particle size distribution was consistent with the data collected for the AlPO_4_ lots over several years, with same overall profile and derived diameters as reported previously [5].

Off-line characterization of AlPO4 was complemented by in-line PAT using probes to determine feasibility of monitoring adjuvant manufacturing in real time. Similar to LD, FBRM data demonstrated no observable differences in particle size distribution between intermediate and final AlPO_4_, manufactured at full scale. The two different particle sizing techniques also yielded similar results (Fig. 3). It is also worth mentioning that in this study the particle size of all AlPO_4_ samples from small-scale precipitation was approximately 25 μm, while all AlPO_4_ lots manufactured at large-scale was approximately 11 μm regardless of particle sizing method. This difference is likely due to the geometry, mixing, and sheer volume of the reactor in large-scale production, however in any case is illustrative of the importance of monitoring such attributes when assessing the impact of major process changes. Size distribution profiles recorded in real time by FBRM® were used to monitor small-scale AlPO_4_ precipitation from the AlCl_3_ and Na_3_PO_4_ raw materials. These profiles were useful to track the reaction progress and mark its completion. In addition to vaccine adjuvant, FBRM® is also an alternative method to LD to measure size distribution of adsorbed drug substances for purposes of product knowledge. The advantages of measuring particle size by in-line versus off-line methods include efficiency in data acquisition and the ability of the technology to monitor reactions in real time. In addition, multiple product and process parameters can be monitored in parallel, for example with an in-line microscopy probe for morphology of the reaction constituents [4]. Furthermore, the ParticleView probe (Mettler Toledo Inc., USA) can be used in combination with the ParticleTrack to monitor the relative backscatter index (RBI) of a reaction (see Fig. S3). RBI is an image-based parameter that indicates how particle size, shape, and concentration are changing in real-time. This is the first time implementing in-line FBRM® and ReactIR probes to visualize the process of AlPO_4_ suspension formation in terms of changes in particle size distribution and IR spectroscopy, i.e., highlighting the potential of this technology to be used in-line for real-time process monitoring.

Because of the importance of ensuring a consistent vaccine adjuvant manufacturing process and product, it is useful to characterize the spectral features of both the intermediate and raw material stages of AlPO_4_ manufacturing. A compositional change was apparent from off-line FTIR and Raman spectroscopy analysis whereby the P—O stretch peak shifted to a higher frequency region from the intermediate to the final AlPO_4_ manufacturing stage (Fig. 1b and c). This shift to higher energy in the peak corresponds to increase in P—O bond strength. A similar finding using Raman and FTIR spectroscopy was reported by Burrell et al. [14].

While consistent results and data trends were observed between in-line and off-line measurements using similar technologies, the in-line techniques offer significant advantages both from a scientific and business perspective. IR spectroscopy using in-line probes was able to detect the same shift in the P—O stretch of AlPO_4_ between the large-scale intermediate and final samples. The shift to greater frequency indicated a stronger phosphate bond in the final AlPO_4_ adjuvant (Fig. 5). In-line spectroscopic methods were able to record single measurements of samples (large-scale AlPO_4_), as well as characterize spectral features during the precipitation of the adjuvant from its raw materials. Additionally, the changes in the mid-IR region of the AlCl_3_ and AlPO_4_ spectra in real time could monitor the reaction progress and determine its endpoint [15]. Similarly, the adsorption of Tetanus Toxoid protein antigen to AlPO_4_ adjuvant could also be measured by monitoring the IR signal for free Amide at 1518 cm^−1^ and adsorbed protein antigen at 1663 cm^−1^ (Fig. 6), consistent with changes in protein secondary structure previously observed by FTIR [5]. In addition to particle size and IR spectral data, in-line probes are also available for measuring other key parameters, such as pH and temperature inside the reactor. Information on the pH is useful during AlPO_4_ precipitation as it can impact the physiochemical properties of the adjuvant [16].

The in-line experiments in this study performed during small-scale AlPO_4_ adjuvant manufacturing were performed to assess the feasibility of applying similar technology to the large-scale process. It is reasonable to expect that major changes in parameters such as process scale may have significant impacts on various product quality attributes, such as AlPO_4_ particle size and P—O bond shift. Therefore, it is important to have analytical tools in place to assess such impacts as process changes are inevitable during the development and life cycle of most biological products. The fact that both the in-line and off-line technologies can readily and consistently detect a major change in the material attributes due to process scale supports the utility of this approach. While the results from in-line experiments were consistent with the off-line results, in-line PAT offers obvious benefits in terms of consistency, efficiency, and cost. The increased use of PAT in vaccine manufacturing can enable such improvements as real-time product release testing and digitization of batch records. Tasks that once required multiple personnel to complete can now be performed automatically in a fraction of the time. Although the focus of this study was on qualitative methods to characterize the changes in the composition and particle size between intermediate and final AlPO_4_ adjuvant during the manufacturing process, these analytical technologies have the potential to be used for quantitative studies such as the Al and P content in AlPO_4_ (observed via XPS), and the concentration of raw materials during AlPO_4_ precipitation to determine saturation and reaction progress.

As described in this paper, in-line testing (PAT) provides several quality and business-related benefits for better analysis of adjuvants and their interactions with developmental and commercially licensed vaccines. Off-line QC testing is slow, often requires significant volume of material for testing and requires extensive maintenance and upkeep of many different analytical instruments. This is also expensive and time consuming. PAT provides fast turnaround time without the need for sampling, allowing manufacturing scientists opportunity to make real-time decisions to better ensure quality and consistency in product manufacturing, including adjuvants. To that end, PAT provides a much faster vehicle for analytical assessment of manufacturing processes during product development, such as fermentation monitoring or consistency during manufacturing of an adjuvant with complex biophysical properties, especially in the presence of the mixture of proteins. Ultimately, the goal of analytical testing is to ensure there is solid data available to characterize and release developmental and commercial products. The work done in this paper demonstrates how PAT can be employed to provide these data in real-time and in a comparable fashion to conventional analytical testing. In conclusion, the combination of biophysical assays and in-line process analysis is a lean solution for meeting the increasing demands for commercialized vaccines.

## 5 Conclusion

The results of this study indicate that, during AlPO_4_ adjuvant manufacturing process, the in-line process analytical technology (PAT) was shown to be useful to monitor particle size distribution (by FBRM®) and P—O bond shift (by IR spectroscopy) in real time in a small-scale reactor while a reaction is in progress. The results support the utility of adopting in-line PAT where feasible in large-scale adjuvant and vaccine manufacturing processes. Biophysical properties of adjuvants are key material attributes and it is informative to monitor them during vaccines manufacturing. Hence, in-line technologies such as those described here have the potential to streamline the development of new adjuvanted vaccines, as well as facilitate efficient life cycle management of marketed products.

## Declaration of Competing Interest

Carmen Mei, Sasmit Deshmukh, Bruce Carpick, Matthew Balmer, Daniel Chapman, Nicole Lazaris, Liliana Sampaleanu, and Marina Kirkitadze are employees of Sanofi Pasteur. Jim Cronin, Shuxin Cong, Ulrich Schacht, and Katherine Drolet-Vives, Moriam Ore and Sylvie Morin have no relevant affiliations or financial involvement with any organization or entity with a financial interest in or financial conflict with the subject matter or materials discussed in the manuscript.

## Appendix A Supplementary data

Supplementary material Image 1
