# Supplementary material for: Aluminum Phosphate Vaccine Adjuvant: Analysis of Composition and Size Using Off-Line and In-Line Tools
Source: Comput Struct Biotechnol J. 2019 Aug 21;17:1184–94. doi: 10.1016/j.csbj.2019.08.003 (PMC6739432; doi:10.1016/j.csbj.2019.08.003)
Supplement: Supplementary file 1 — Supplementary material [file mmc1.docx]

**Supplementary Information**

**Aluminum phosphate vaccine adjuvant: Analysis of composition and size using off-line and in-line tools**

Carmen Mei, Sasmit S. Deshmukh, Jim Cronin, Shuxin Cong, Daniel Chapman, Nicole Lazaris, Liliana Sampaleanu, Ulrich Schacht, Katherine Drolet-Vives, Moriam Ore, Sylvie Morin, Bruce Carpick, Matthew Balmer, Marina Kirkitadze





**Figure S1.** Raman spectra of solid Na_3_PO_4_ (gray trace) and solid AlCl_3_ (brown trace). Na_3_PO_4_ showed prominent peaks for the phosphate group at 412 cm^-1^, 548 cm^-1^, and 942 cm^-1^. AlCl_3_ showed AlCl_3_ stretch at 560 cm^-1^, Al‒O stretch at 425 cm^-1^, and hydrated AlCl_3_ at 520 cm^-1^.


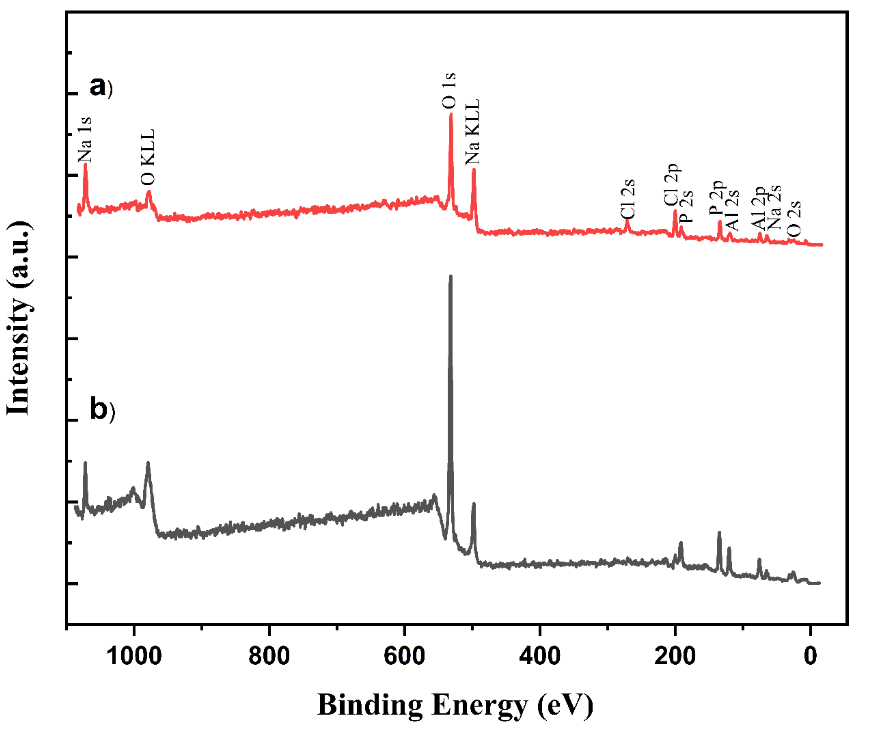


**Figure S2.** XPS survey spectra for intermediate (a) and final (b) AlPO_4_ adjuvant.

Table S1: XPS determination of atomic percent of elements for the intermediate and final AlPO_4_ samples, using two different electron take-off angles. P/Al ratio was consistent with previously reported value obtained by NMR for liquid AlPO_4_ samples (6).

| **Samples** | **C** | **O** | **Na** | **Al** | **P** | **Cl** | **P/Al** |
| --- | --- | --- | --- | --- | --- | --- | --- |
| (Intermediate) AlPO_4_ at 15^o^ take-off angle | 8.0 | 42.9 | 20.2 | 9.9 | 9.4 | 9.6 | 0.95 |
| (Final) AlPO_4_ at 15^o^ take-off angle | 2.8 | 58.3 | 9.6 | 13.7 | 13.4 | 2.3 | 0.98 |
| (Intermediate) AlPO_4_ at 45^o^ take-off angle | 7.0 | 43.3 | 20.5 | 9.6 | 9.7 | 9.9 | 1.0 |
| (Final) AlPO_4_ at 45^o^ take-off angle | 0.9 | 60.8 | 9.7 | 13.7 | 13.3 | 1.7 | 0.97 |

**Table S2:** XPS determination of maximum binding energies values of the spectral peaks for intermediate and final AlPO_4_ adjuvant samples, using two different electron take-off angles.

| Samples | Al 2p (FWHM),  eV | P 2p (FWHM),  eV | O 1s (FWHM),  eV |
| --- | --- | --- | --- |
| AlPO_4_ (Intermediate ) at 15^o^ take-off angle | 74.41 | 133.79 | 531.10 |
| AlPO_4_ (Final) at 15^o^ take-off angle | 75.74 | 135.40 | 532.32 |
| AlPO_4_ (Intermediate) at 45^o^ take-off angle | 74.62 | 133.69 | 531.51 |
| AlPO_4_ (Final) at 45^o^ take-off angle | 75.23 | 134.46 | 532.04 |


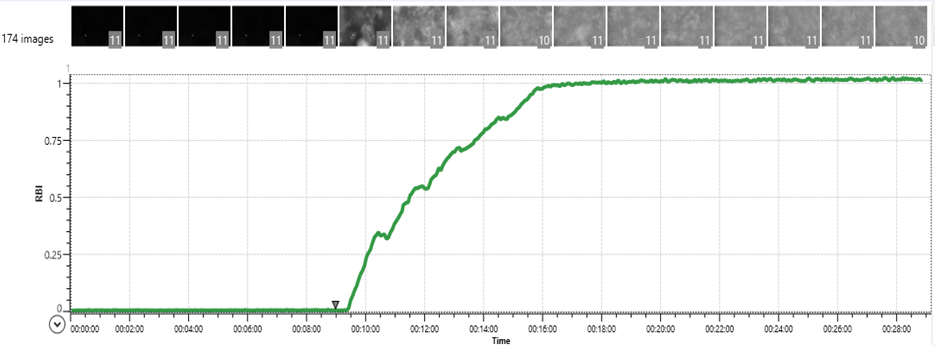


**Figure S3.** FBRM: Real-time analysis of Relative Backscatter Index (RBI) during a small scale study AlPO_4_ precipitation from raw materials. Data presented are one of four runs.


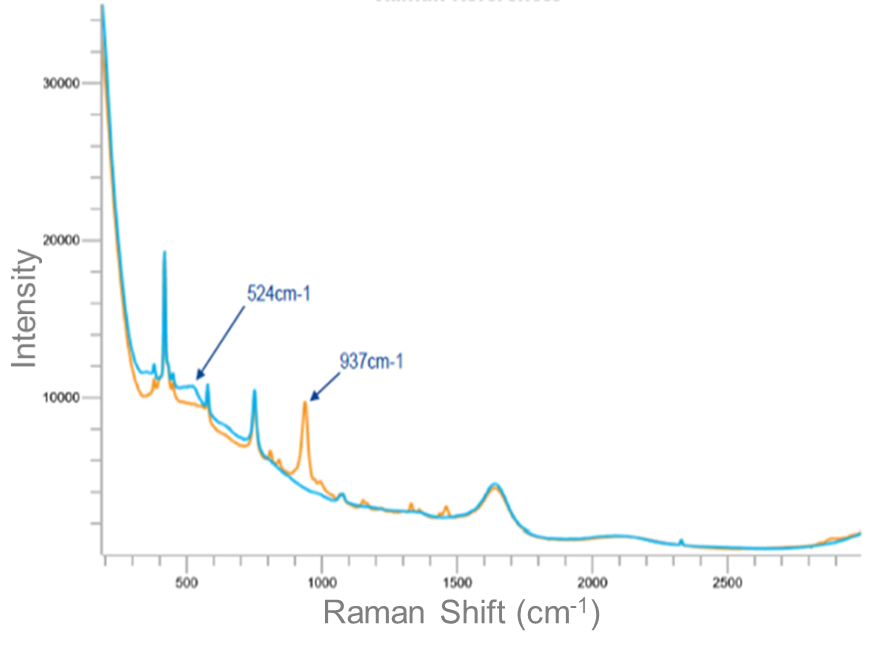


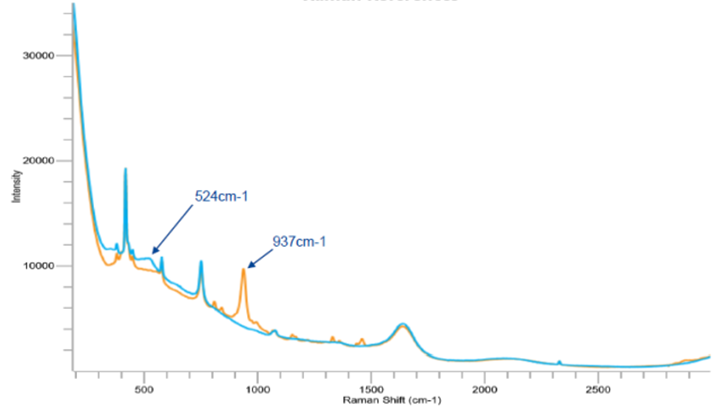


Raman Shift (cm^-1^)

Intensity


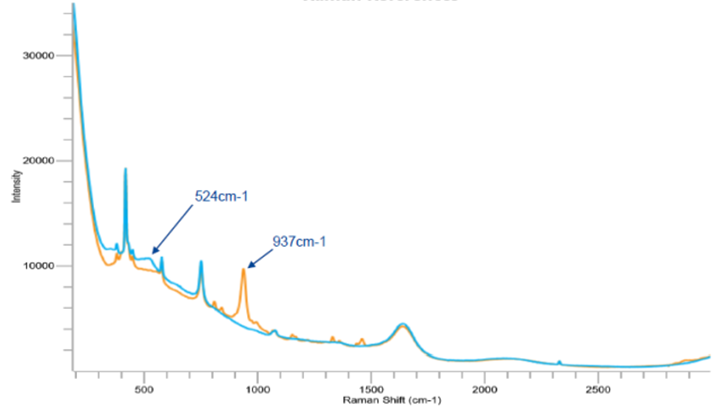


Raman Shift (cm^-1^)

Intensity


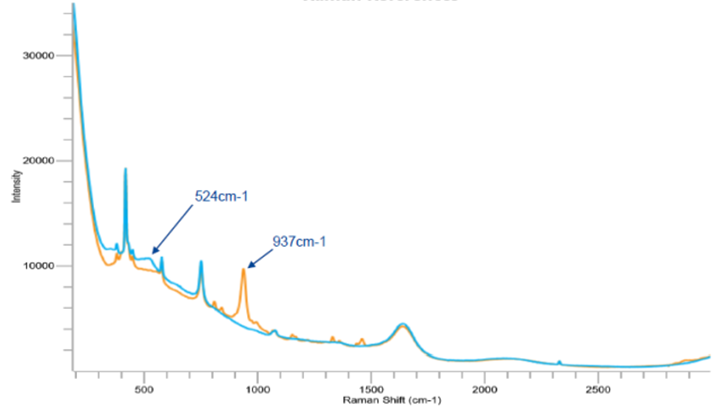


Raman Shift (cm^-1^)

Intensity

**Figure S4**. Raman spectral overlay of AlPO_4_ raw materials solutions AlCl_3_ (blue) and Na_3_PO_4_ (orange) salt solutions from inline ReactRaman 7 inline probe.
